# Editorial: Adenosine pathways in cancer immunity and immunotherapy

**DOI:** 10.3389/fimmu.2023.1298487

**Published:** 2023-10-10

**Authors:** Junjiang Fu, Luca Antonioli, Ali H. El-Far

**Affiliations:** ^1^ Key Laboratory of Epigenetics and Oncology, the Research Center for Preclinical Medicine, Southwest Medical University, Luzhou, Sichuan, China; ^2^ Department of Clinical and Experimental Medicine, University of Pisa, Pisa, Italy; ^3^ Department of Biochemistry, Faculty of Veterinary Medicine, Damanhour University, Damanhour, Egypt

**Keywords:** adenosine, cancer, immunity, immunotherapy, pathways

Adenosine signalling represents a critical metabolic pathway involved in regulating tumour immunity, being co-opted by tumours to promote their growth, and impair immunity. Adenosine is produced at high tumour microenvironment (TME) levels in response to hypoxia. It is a broadly immunosuppressive metabolite that regulates innate and adaptive immune responses. Inhibition of adenosine-generating enzymes represents one strategy for promoting antitumor immunity by enhancing T cell and NK cell functionality and suppressing the pro-tumorigenic effects of myeloid cells and other immunoregulatory cells. Research into immunotherapeutic targeting various aspects of adenosine signalling is already underway, with several agents counteracting the adenosine axis have been developed. Pre-clinical studies have demonstrated anti-tumour activity alone and in combination with other immunotherapies, though more research is needed to understand their viability as a treatment option.

Extracellular adenosine activates cellular pathways through one of four known G-protein-coupled adenosine receptors: A_1_, A_2A_, A_2B_, and A_3_. The A_2A_ receptor is a high-affinity receptor expressed on T cells and natural killer T (NKT) cells, monocytes, macrophages, DCs, and natural killer (NK) cells. In contrast, the A_2B_ receptor is a relatively low-affinity receptor most highly expressed by macrophages and DCs (1). Many factors that favour adenosine generation-tissue disruption, hypoxia, ectonucleotidase expression, and inflammation-are highly characteristic of TME. Significant work has thus been done in targeting various aspects of tumour-associated adenosine signalling to enhance the immune response to malignancy (2).

Adenosine is an immunosuppressive metabolite produced at high levels within TME. Hypoxia, increased cell turnover, and expression of CD39 and CD73 are essential factors in adenosine production. Adenosine pathway blockade in immunotherapy for cancer is of great importance for cancer patients. Targeting of the adenosine pathway has generally focused on two primary aspects of immunosuppressive adenosine through (1) inhibition of adenosine production in the TME through targeting CD73 and CD39 and (2) the blockade of adenosine signalling through targeting the A_2A_ and A_2B_ receptors (3). Therefore, targeting the A_2B_ receptor as an immunotherapeutic target in pancreatic cancer (Strickland et al.).

Combined with novel biomarkers, immune checkpoint inhibition may provide alternative pathways for treating chemotherapy-resistant triple-negative breast cancer (TNBC). Adenosine A_2A_ receptor is associated with aggressive clinical outcomes and reflects an immunosuppressive TME in human breast cancer. Also, zoledronate, the standard of care for high-risk early breast cancer patients, -induced growth inhibition and enhanced B and T lymphocyte infiltration into the orthotopic tumours with down-regulated CD73 (Petruk et al.). Because CD155 and CD73 expression was associated with a poor response to NAC and poor prognosis in this chemotherapy-resistant TNBC cohort, supporting additional immune checkpoint receptor inhibitor therapy (Cabioglu et al.).

Gastric cancer (GC) is one of the most common malignancies and a leading cause of cancer-related deaths worldwide. GC patients are usually in the advanced stage at first diagnosis and miss the best opportunity for treatment. The accumulation of extracellular adenosine inhibits the normal function of immune effector cells and facilitates the effect of immunosuppressive cells to enhance GC cell proliferation and migration. Wang et al. provided a comprehensive review that adenosine signalling can be an optimal target for GC immunotherapy.

The clinical benefit of immune checkpoint blockade in cancer therapy and the promising preclinical activity of adenosine pathway blockade is pivotal for cancer therapy. Several agents that block distinct targets along the adenosinergic pathway are presently in early-phase clinical trials.

Zohair et al. found that A_2A_ receptor could be a promising therapeutic target to overcome immune evasion prevailing within the TME of breast cancer patients. We encourage researchers to investigate the blockage of natural bioactive compounds to adenosine pathways in preclinical and clinical phases due to their safety, margine, and anticancer benefits.

## Author contributions

JF: Writing – original draft, Writing – review & editing. LA: Writing – review & editing. AE-F: Writing – original draft, Writing – review & editing.
